# Tailoring plant-based food emulsions with oil bodies and proteins recovered by alkali aqueous extraction from rapeseed (*Brassica napus* L.) oilseeds

**DOI:** 10.1016/j.crfs.2026.101596

**Published:** 2026-10-14

**Authors:** Hanitra Rabesona, Christelle Lopez

**Affiliations:** INRAE, UR BIA, Nantes, F-44316, France

**Keywords:** Oil body, Oleosome, Lipid droplet, Natural oil-in-water emulsion, Interface, Homogenization, Plant based emulsion

## Abstract

Oil bodies (OBs) and storage proteins recovered from oilseeds by aqueous extraction are promising ingredients for the development of plant-based food emulsions. This study aimed to co-extract natural OBs and storage proteins from rapeseed oilseeds, to explore the microstructure, to analyse the composition, to investigate the emulsifying properties of the proteins and to determine the impact of pH on the physical stability of natural OBs, homogenized OBs, proteins and protein-stabilized oil droplets in emulsions. Alkali aqueous extraction at pH 9.5 enabled the recovery of well-dispersed OBs together with the major storage proteins, cruciferin and napin. The mean diameter of rapeseed OBs was 1.4 ± 0.1 μm. They were surrounded by a layer of phospholipids (0.27 wt% of total lipids) and also contained phytosterols (8628 ± 340 mg/kg lipids; 44.2 wt% β-sitosterol), tocopherols (513 ± 27 mg/kg; 59.1 wt% γ-tocopherol) and carotenoids (66.0 ± 2.4 mg/kg lipids; 71.6 wt% lutein). Protein analyses showed that napin preferentially adsorbed at oil/water interfaces, whereas cruciferin remained mainly in the continuous phase andwas involved in oil droplet aggregation below pH 7 owing to decrease in cruciferin solubility and changes in interfacial interactions. This work demonstrates that the functional properties of rapeseed aqueous extracts are governed not only by the intrinsic characteristics of native OBs but also by their interactions with co-extracted storage proteins. These findings provide new insight into the rational design of sustainable plant-based emulsions and support the valorization of rapeseed aqueous extracts as multifunctional food ingredients.

## Highlights

• Rapeseed oil bodies and storage proteins (napin, cruciferin) were co-extracted in alkaline conditions.
• Napin dominated interfacial adsorption, while cruciferin promoted aggregation below pH 7.
• Homogenization and pH governed interfacial organization and emulsion stability.
• Oil body–protein interactions drive emulsion functionality and physical stability.

## Graphical abstract

**Figure:**
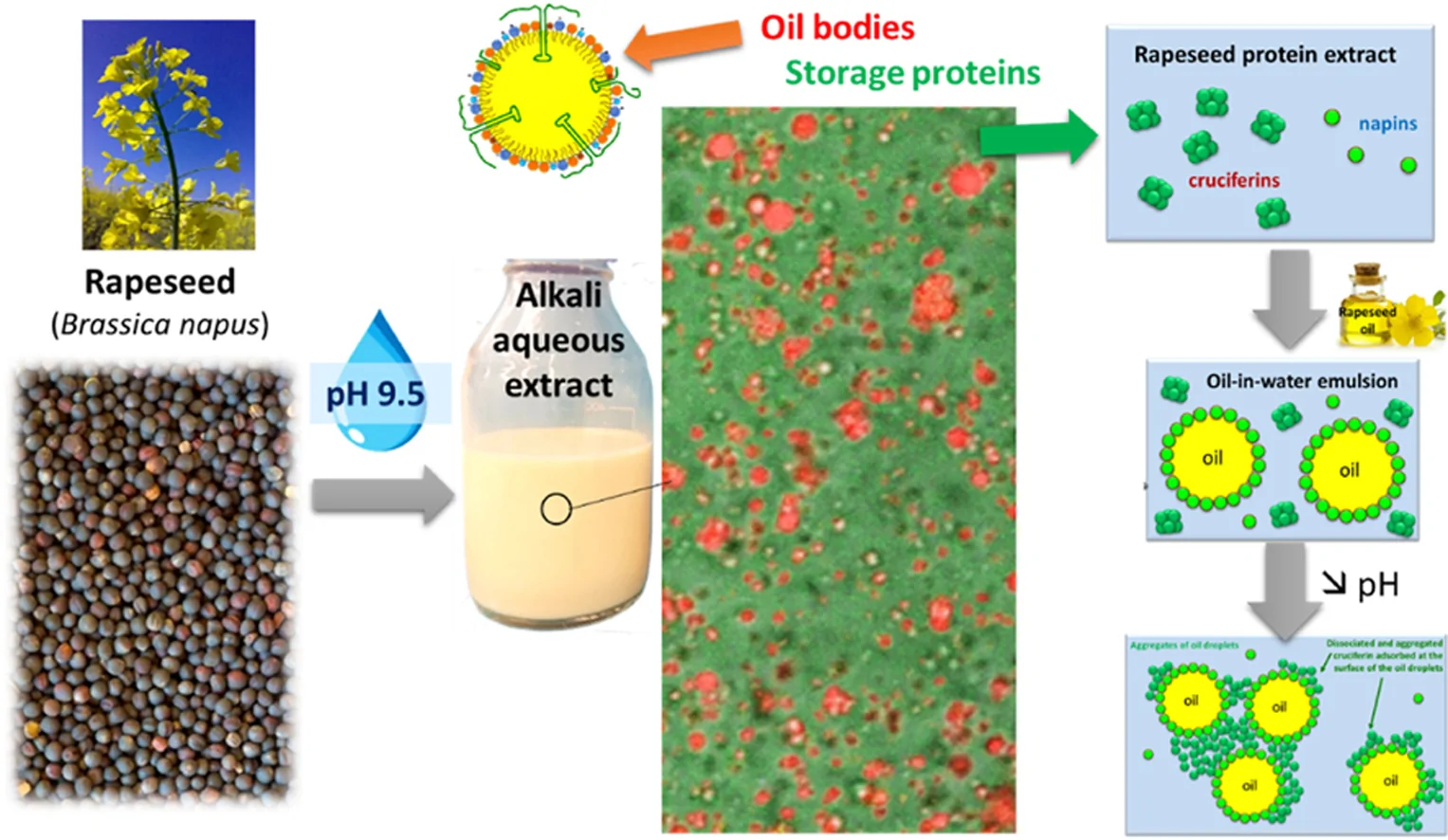

## 1 Introduction

With the ever-increasing consumer demand worldwide for sustainable, natural, healthy and plant-based foods, one of the major challenges in the food industry is to replace synthetic and animal-origin ingredients with plant-based ingredients in some formulations (Tang and Ghosh, 2023). Partial substitution will favour rational combination of plant-based and animal-derived ingredients in the diet. Among new and developing plant sources for food applications, proteins and oil bodies (OBs, also known as lipid droplets or oleosomes (Nikiforidis, 2019a)) from oilseeds deserve particular consideration. Moreover, many foods are oil-in-water emulsions (drinks, mayonnaise, sauces). Boosting the development of food emulsions based on plant OBs and on plant protein emulsifiers by exploring the biodiversity of oleaginous seeds is therefore in the spotlight of many food scientists (Şen et al., 2024; Nikiforidis et al., 2014; Romero-Guzmán et al., 2020a; Lopez et al., 2021, 2023a, 2023b; Guan et al., 2025). In this context, a better knowledge and understanding of the functional properties of plant OBs and proteins recovered from oilseeds of nutritional interest such as rapeseeds are required to develop innovative plant-based food emulsions.

Rapeseed (Brassica napus) is the second-largest cultivated oilseed crop in the world and is a major crop of special environmental and nutritional interests. The demand for edible plant oil has been the economic driver for rapeseed cultivation for decades. Currently, rapeseed is mainly cultivated worldwide for its healthy seed oil that is consumed in various foods and the protein-rich rapeseed meal is mainly used for animal feeding.

In the last ten years, plant OBs recovered by aqueous extraction from various oilseeds have gradually attracted the interest of more researchers and have received increasing amounts of attention in the food industry (Shi et al., 2024). The microstructure, composition and physico-chemical properties of plant OBs recovered from various crops have been investigated for food applications, e.g., OBs from hemp seeds (Lopez et al., 2021; Garcia et al., 2021), camelina seeds (Lopez et al., 2023a), chia seeds (Lopez et al., 2023a), sunflower seeds (Yang et al., 2023), rapeseed oilseeds (Romero-Guzmán et al., 2020a). OBs have attracted attention as food-grade ingredients in formulations e.g. in plant-based mayonnaise-like emulsion (Romero-Guzmán et al., 2020a), for their intrinsic emulsifying properties, and potentialities for encapsulation.

The extraction of rapeseed OBs for food formulations has been considered (Romero-Guzmán et al., 2020a, 2020b, 2020c). However, their composition, structure and physico-chemical properties need to be explored in more detail in order to enrich the understanding of their nutritional value and their colloidal stability in the complex environment of food products. As demonstrated in the case of plant OBs suspensions recovered by aqueous extraction from various oilseeds and walnuts, the colloidal stability depends on pH and ionic environment (i.e., salts such as NaCl, CaCl_2_) with consecutive electrostatic repulsion forces (Lopez et al., 2021, 2023a, 2023b; Romero-Guzmán et al., 2020b). Moreover, upon processing, natural OBs can be submitted to high shear stress or to high pressure homogenization. Understanding the preferential adsorption of rapeseed storage proteins at the surface of oil droplets and investigating their potentialities as plant emulsifiers to efficiently guaranty the physical stability of emulsions will help in developping new emulsion-based food formulations.

Rapeseed proteins are recognized for their balanced amino acid profile and for providing all essential amino acids (Wanasundara et al., 2016a). They could contribute to satisfy the world protein demand that is rising in parallel with the growing population. Rapeseed proteins also have interesting functional properties such as emulsifying properties (Tang and Ghosh, 2023). However, less than 1 wt% of the global plant-based protein market is taken up by rapeseed proteins and they are therefore underutilized (Tang and Ghosh, 2023). The major proteins of rapeseeds are cruciferin (11S hexameric globulin, MW 300 kDa) and napin (2S albumin, MW 12 – 17 kDa) that comprise 60 wt% and 20 wt%, respectively, of the total proteins in mature seeds. In the last years, extent of research on rapeseed proteins for food emulsions has demonstrated an increased interest. Rapeseed proteins recovered by aqueous extraction tpgether with OBs could play a major role as natural food-grade plant-based emulsifiers to prepare and stabilize oil-in-water emulsions such as those present in many food products (e.g., mayonnaise, beverages, coffee creamer, salad dressings, spreadables, meat products). However, few research studies investigated the emulsifying properties of rapeseed proteins recovered by alkali aqueous extraction (Ntone et al., 2021).

The stability of emulsion lipid droplets and natural OBs is a key factor in achieving their commercialization, as their physical instability can lead to a decrease in their functionality, and any product requires a certain shelf life. Due to the complexity of food systems, understanding the physical stability of plant proteins, oil droplets in emulsions and natural OBs suspensions under varying pH conditions is therefore crucial for their application in the food industry.

This study aims at filling key knowledge gaps that concern the valorization of aqueous extracts recovered from rapeseed oilseeds containing OBs and storage proteins (cruciferin and napin) to prepare innovative plant-based food formulations. The objectives were i) to recover OBs and proteins from rapeseed oilseeds using an integrated green process based on aqueous extraction, ii) to examine the microstructure and composition of rapeseed OBs, iii) to explore the emulsifying properties of rapeseed proteins, iv) to investigate the changes in the size and surface composition of OBs induced by high-pressure homogenization, and v) to determine the impact of pH on the physical stability of rapeseed OBs, proteins, emulsions containing rapeseed protein coated oil droplets and homogenized rapeseed OBs.

## 2 Materials and methods

### 2.1 Materials

**Rapeseed oilseeds.** Rapeseed (*Brassica napus* L.; variety named Allize) oilseeds were kindly provided by IGEPP unit (INRAE, Le Rheu, France). According to the provider, the rapeseed oilseeds were food grade as they did not contain euricic acid and had a low glucosinolate content (12 ± 1 mg/100 g). The composition of the rapeseed oilseeds as provided was as follows: 9.0 ± 1.2 wt% moisture, 37.0 ± 1.1 wt % of oil and 19.0 ± 0.5 wt % of protein in wet basis.

**Rapeseed oil.** Organic rapeseed oil was produced by the Huilerie Vigean (36700 Clion-Sur-Indre, France) and purchased at the Biocoop store (Landivisiau, France).

### 2.2 Processing

#### 2.2.1 Alkali aqueous extraction from rapeseeds

The internal cellular material of rapeseed oilseeds, i.e. oil bodies and proteins, were recovered by aqueous extraction using various experimental parameters. All extractions were performed in batches of 200 g of rapeseed oilseeds. First, the aqueous extraction was performed in ultrapure water as previously described for other seeds (Lopez et al., 2021, 2023a) and walnuts (Lopez et al., 2023b). Then, we followed the extraction method proposed by Gray's group (De Chirico et al., 2018) with some modifications. The alkali aqueous media was prepared with pipes buffer (1,4-piperazinediethane sulfonic acid; purity > 99 %; Sigma) in purified water (Milli-Q waters) to reach a concentration of 10 mM. The pH of the pipes buffer was adjusted to pH 9.5 with NaOH (1.0 mol/L). The presence of 100 mM NaCl (analytical grade from Sigma-Aldrich, St Louis, MO, USA) in the alkali solution was tested. The sequential refinery process employed in this study to recover alkali aqueous extracts from rapeseed oilseeds is presented Fig. 1. Whole rapeseeds were soaked (seeds/alkali aqueous media 1:3 wt/wt) for 8 h at 4 °C. After soaking, the solid/solvent ratio was adjusted to 1:7 wt/v and the dispersion of the seeds in the alkali aqueous media was ground for 3 min at a speed of 6000 rpm (Turbomix plus, Moulinex, France) to disrupt the cell walls and release the cellular material. The resulting slurry was then filtered through a layer of cheesecloth to remove the external part of the seeds (e.g. rapeseed shells) and cell wall components. The alkali aqueous extract containing the rapeseed OBs and plant storage proteins was recovered in the filtrate. The whole rapeseed alkali aqueous extract is a natural oil-in-water emulsion that contains OBs and plant storage proteins that were separated by centrifugation (Eppendorf® 5810R centrifuge, Merck KGaA, Darmstadt, Germany). The whole rapeseed alkali aqueous extract was centrifuged at 1000 *g* for 30 min at 15 °C to isolate the OBs without inducing their coalescence. The layer of OB-rich cream at the top of the tubes was manually collected. Centrifugation of the whole rapeseed alkali aqueous extract was performed at 7000 *g* for 30 min at 15 °C in order to remove OBs in the cream phase (without damage of their physical integrity) and to collect the lower protein-rich phase, as already described (Lopez et al., 2024). The layer of OB-rich cream at the top of the tubes was manually collected. This step was repeated twice. The defatted aqueous extracts (fat content below 0.3 g/100 g) contained rapeseed oilseed storage proteins and were used for experiments without any further purification of the rapeseed proteins.

**Fig. 1:**
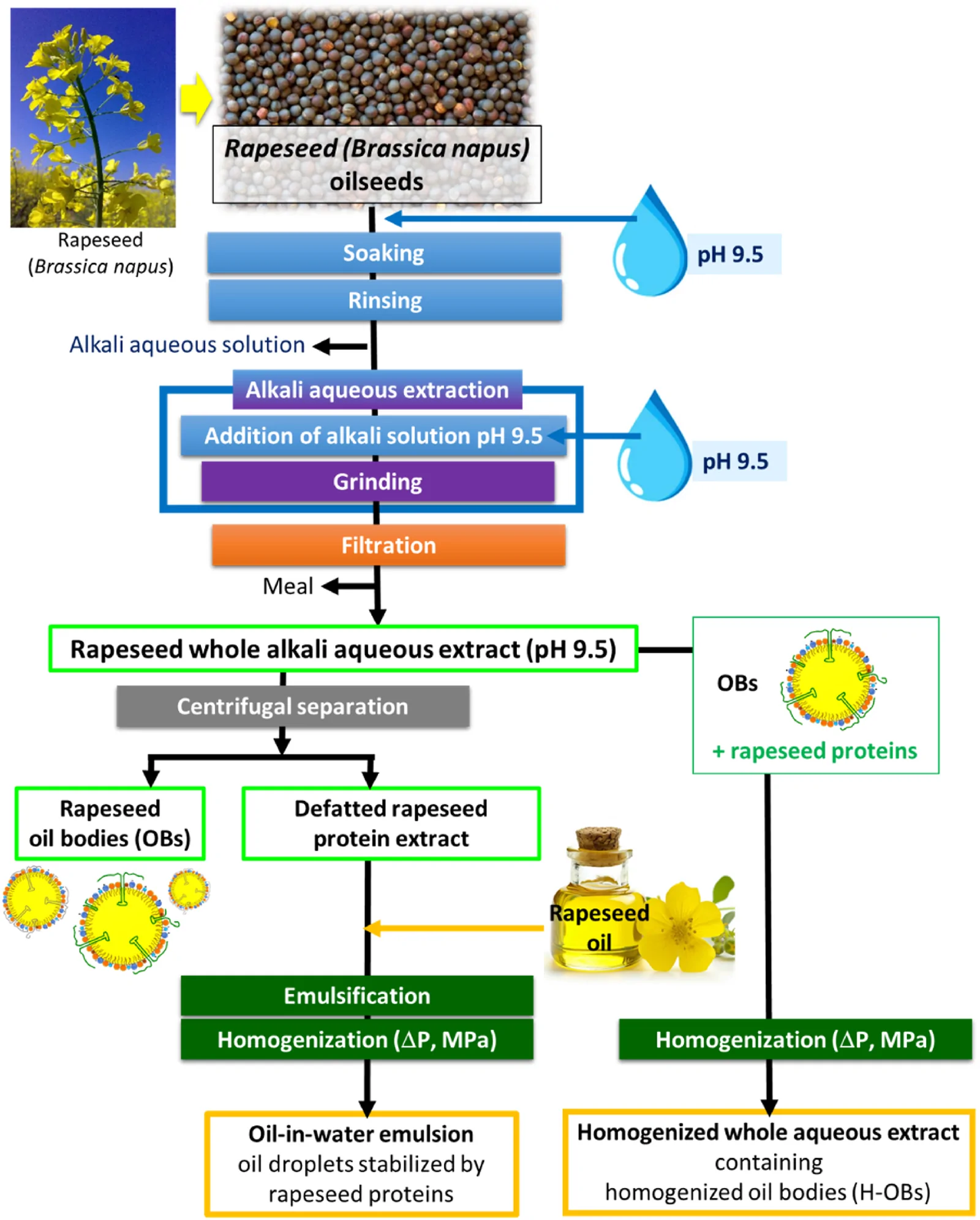
Schematic diagram of the integrated refinery process used for the alkali aqueous extraction of oil bodies (OBs) and storage proteins from rapeseed oilseeds. The bottom part of this schematic diagram presents the homogenization of the whole aqueous extracts, the use of centrifugal separation to recover OBs and defatted rapeseed protein extracts and the preparation of oil-in-water emulsions stabilized by the rapeseed proteins.

To prevent the growth of bacteria, sodium azide (NaN_3_, 0.01 g/100g) was added to the whole aqueous extracts and to the defatted protein extracts.

#### 2.2.2 Preparation of oil-in-water emulsions and homogenized whole aqueous extracts

Oil-in-water emulsions were prepared with the defatted protein alkali aqueous extracts recovered after centrifugation of the whole aqueous extract and removal of the OBs (Fig. 1). The defatted protein alkali aqueous extracts were adjusted to pH 9.5 and pH 7.5 (HCl or NaOH solutions). The amount of rapeseed oil necessary to prepare an emulsion with 3 g/100g oil content was mixed with the protein extract (e.g., for 200 g emulsion: 6 g oil, weight adjusted to 200 g with the protein aqueous extract). The emulsions were prepared using a multi-step process including emulsification by high-shear mixing with a Polytron (25000 rpm, 30 s), followed by homogenization at 4 MPa with a laboratory high-pressure homogenizer (PandaPlus 1000, GEA Niro Soavi, Italy) (Fig. 1). The emulsions were recovered and analyzed. The emulsions at pH 9.5 and pH 7.5 were prepared three times from 3 independent defatted protein alkali aqueous extracts.

Homogenized whole aqueous extracts were prepared by passing the whole rapeseed aqueous extracts (pH 9.5) through a two-stage laboratory high-pressure homogenizer (PandaPlus 1000, GEA, Italy). Homogenization was performed at 2.5, 5, 10, 30 and 60 MPa. The homogenized whole rapeseed aqueous extracts were recovered and analyzed (Fig. 1). The homogenization of the whole aqueous extracts at various pressures was performed from three independent whole aqueous extracts recovered from rapeseed oilseeds.

#### 2.2.3 Changes in the pH and investigation of the physical stability of dispersions

The pH of the samples (i.e. suspensions of OBs, defatted protein extracts, oil-in-water emulsions and homogenized whole aqueous extracts) was adjusted using 0.1 M HCl or NaOH solutions. The samples were stored in 15 mL plastic tubes to evaluate their physical stability as a function of pH at the macroscopic scale, as previously performed (Lopez et al., 2021, 2023a, 2023b). Pictures of the tubes were taken to characterize the physical stability of the samples as a function of pH. The experiments dedicated to the impact of pH on the physical stability of the samples were performed three times from three independent preprarations of the samples (i.e. suspensions of OBs, defatted protein extracts, oil-in-water emulsions and homogenized whole aqueous extracts). A representative picture of the tubes containing the samples at various pH values is presented in the paper.

### 2.3 CLSM

The microstructure of whole rapeseed aqueous extracts, OBs, defatted protein extracts, homogenized OBs and emulsions was examined by confocal laser scanning microscopy (CLSM). A microscope NIKON A1R (NIKON, Champigny sur Marne, France) equiped with a x60 oil immersion objective was used. Several fluorescent dyes were used. The lipid-soluble Nile Red fluorescent probe (5H-benzo-alpha-phenoxazine-5-one,9-diethylamino; supplied by Sigma Aldrich, St Louis, USA; 100 μg/mL in propanediol) was used to stain the TAG-rich core (excitation wavelength = 560 nm). Fast Green FCF (Sigma-Aldrich, St. Louis, USA; 10 mg/mL in water) was used to stain proteins (excitation wavelength = 636 nm). The fluorescent head group-labelled phospholipid analogue Lissamine rhodamine B sulfonyl dioleoylphosphatidylethanolamine (Rh-DOPE, 1 mg/mL; Avanti Polar lipids Inc., Birmingham, England) was used to investigate the lateral distribution of phospholipids on the surface of OBs (excitation wavelength = 560 nm). The samples were kept at room temperature for at least 30 min prior to observation by CLSM.

### 2.4 Particle size measurements

Laser light scattering was used to determine the size distributions of oil droplets in various samples: OBs recovered in the alkali aqueous extracts, homogenized OBs and emulsion oil droplets prepared from rapeseed oil and defatted protein extract. For measurements, the samples were diluted in buffer as appropriate. Each sample was diluted 1/10 vol/vol in SDS 1 % wt/v to investigate the possible presence of particle aggregation by the comparison of the results in presence or absence of SDS. The apparatus was a laser diffraction analyzer (Horiba LA-960V2, Retsch Technology, Germany). The refractive indices used were 1.47 for the dispersed phase (OBs or rapeseed oil) and 1.33 for the continuous phase (buffer adjusted in pH, pH 9.5 or pH 7.5). Each sample was prepared at least in triplicate (n = 3). The particle size measurements of each sample were performed in triplicate using three independent aliquots of the sample (for each kind of sample, the number of size measurements corresponded at least to 3 x 3 = 9 values; the results are given as mean ± stand deviation).

In order to examine the potential changes in oil droplet size during the storage at 4 °C for 3 weeks of the emulsions prepared at pH 9.5 and pH 7.5 from rapeseed oil and defatted protein extract, size measurements were performed each two or three days (10 values as a function of time; without SDS and with SDS).

### 2.5 Zeta potential measurements

Dispersions of rapeseed OBs and homogenized OBs were diluted in solutions adjusted at various pHs in the range from 11.5 to 2.5 using NaOH or HCl (dilution: 100 μL OB dispersion in 10 mL solution). About 1 mL of each diluted OB dispersion was filled into a cuvette, which was then placed into the chamber of a Zetasizer Nano ZS (Malvern, Germany) and equilibrated before measurements for 5 min at 20 °C. The zeta potential (ζ) was calculated from the electrophoretic mobility of the OBs and homogenized OBs by applying Henry's equation with the Smoluchowski approximation. The measurements were run five times at 20 °C on at least three independent and freshly prepared samples (at least n = 15 values for each sample).

### 2.6 Lipid analysis of rapeseed OBs

#### 2.6.1 Extraction and quantification of total lipids

For the extraction and quantification of total lipids, the protocol of the cold extraction procedure developed by (Folch et al., 1957) was used. The extraction of total lipids was performed in triplicate, from each sample.

#### 2.6.2 Polar lipid analysis

The quantification of total phospholipids and the determination of the individual phospholipid classes in rapeseed OBs were performed using HPLC combined with an evaporative light scattering detector as previously described for hemp, chia and camelina seed OBs (Lopez et al., 2021, 2023a, 2023b).

#### 2.6.3 Fatty acid profile

The fatty acid profile of rapeseed OBs was determined by a gas chromatography (GC) procedure after methylation with cold methanolic solution of potassium hydroxide, as previously described (Lopez et al., 2021, 2023a, 2023b). The FAME were identified using a mixture of methyl esters as external standard (Mixture ME 100, Larodan, Sweden). The GC analysis was performed in triplicate for each Folch extract of total lipids from rapeseed OBs.

#### 2.6.4 Quantification of total and individual sterols

The identification and quantification of total sterols were performed using gas chromatography with flame ionization (GC-FID) according to (ISO-12228), as previously described (Lopez et al., 2021). The different plant sterols were identified by comparing the retention times with the individual standards (Sigma-Aldrich). The internal standard 5α-cholestan-3β-ol (Sigma D6128) was used for quantification. From rapeseed OBs lipid extracts (n = 9), the analysis of sterols was performed in triplicate; the results presented correspond to the mean of n = 27 values.

#### 2.6.5 Quantification of total and individual carotenoids

The identification and quantification of the carotenoids was performed as in (NF-EN-12823-2), as previously described (Lopez et al., 2021, 2023a). The saponification of the rapeseed OBs lipid extracts was performed as for sterol analysis. The carotenoids were extracted from the KOH-methanolic phase and were analyzed by HPLC (column C18 Vydac 250 × 4 mm) in isocratic conditions and detected at 450 nm. The individual carotenoids were identified by using external standards. For quantification, the external standard beta-carotene (Sigma-Aldrich) was used. From rapeseed OBs lipid extracts (n = 9), the analysis of carotenoids was performed in triplicate; the results presented correspond to the mean of n = 27 values.

#### 2.6.6 Quantification of total and individual tocopherols

The quantification of total and individual tocopherols was performed on Folch lipid extracts from rapeseed OBs according to (ISO 9936:2016), as previously described (Lopez et al., 2021, 2023a). Tocopherols standards (purity > 95 %) were provided by Sigma-Aldrich (St. Louis, MO). HPLC analysis was performed using the 1290 Agilent System (Massy, France) equipped with a C18 column (250 mm × 4.6 mm i.d., 5 μm, HALO(R)-5 column, AMT, Wilmington, Delaware, USA) and a fluorescence detector (296 nm for excitation and 330 nm for emission). The mobile phase consisted of ethanol/methanol (40:60 v/v) in isocratic conditions. The temperature of the column was maintained at 25 °C and the flow rate was 0.8 mL/min. Each sample was analyzed in triplicate. From rapeseed OBs lipid extracts (n = 9), the analysis of tocopherols was performed in triplicate; the results presented correspond to the mean of n = 27 values.

### 2.7 Protein analysis

#### 2.7.1 Determination of the protein profiles: gel electrophoresis

Sodium dodecyl sulfate polyacrylamide gel electrophoresis (SDS-PAGE) was used to determine the protein profiles in the aqueous extracts recovered from rapeseed oilseeds, and the interfacial protein composition of oil bodies (OBs), homogenized OBs (H-OBs) and emulsion oil droplets.

#### 2.7.2 Preparation of the oil droplets

Washing of OBs, homogenized OBs and emulsion oil droplets was performed to remove non-adsorbed proteins from the O/W interface. Briefly, 5 g of emulsion were added and mixed with 5 g of a solution containing 50 wt% saccharose (pH 9.5 or pH 7.5 or pH 7.0). Then, 10 mL of the treated emulsion were deposited under 30 mL of a solution of containing 5 wt% saccharose (pH 9.5 or pH 7.5 or pH 7.0), in 45 mL plastic centrifuge tubes. A centrifugation of the tubes was performed with adapted parameters as a function of the size of the oil droplets to form a layer of washed oil droplets. The tubes were centrifuged at 2000 *g* during 20 min at 15 °C for rapeseed OBs, at 4000 *g* during 20 min at 15 °C for the homogenized whole aqueous extracts (10, 30 and 60 MPa), and at 3000 *g* during 20 min at 15 °C for the emulsion oil droplets prepared at 4 MPa. The washed OBs, H-OBs and emulsion oil droplets recovered at the top of the tubes were used for SDS-PAGE analysis.

#### 2.7.3 Gel electrophoresis

The protein profiles of the samples were characterized by SDS-PAGE with Mini-Protean TGX Precast Gels gradient 4 – 15 % using Mini-Protean Tetra Cell system (Bio-Rad Life Science, France). All reagents were from Bio-Rad Life Science. The protein profiles of the samples were examined in denaturing and either reducing or non-reducing conditions. For the denaturing and reducing conditions, the samples were diluted in 2x Laemmli denaturing sample buffer in reducing conditions with β-mercaptoethanol 5 % (BME, a reducing agent that breaks down the disulfide bonds linkages). For the denaturing and non-reducing conditions, the samples were diluted in 2x Laemmli denaturing sample buffer without BME. The migration buffer was composed as follows: 25 mM Tris, 192 mM glycine, 0.1 % SDS, according to the protocol described by (Laemmli, 1970). The samples were heated at 100 °C for 5 min. Each protein sample was then loaded on a sample well of the gradient gel. Molecular weight (MW) protein marker from MW 10 to 250 kDa (Precision Plus Protein Standards, All Blue, Bio-Rad Life Science, France) was used for MW calibration. The migration was carried out at 200 V for 45 min. The gels were stained with Coomassie Brilliant Blue G-250 staining solution for 2h, under gentle agitation according to (Lawrence and Besir, 2009). Then, each gel was rinsed with distilled water. The gels were then scanned on a flatbed scanner (Image Scanner iii, GE Healthcare Europe, Velizy- Villacoublay, France).

### 2.8 Solubility profile of rapeseed proteins as a function of pH

The solubility profiles of rapeseed proteins recovered in the defatted aqueous extracts were evaluated at pHs ranging from 2.0 to 10.0, as previously performed for camelina seed proteins and described in (Lopez et al., 2024). A defatted protein aqueous extract (25 g) was adjusted at different pHs denoted pHx, with x ranging from 2 to 10. The samples were magnetically stirred at 20 °C for 1 h. The pH was checked and adjusted if required. Each sample at pHx was distributed into two tubes: a first tube (15 g) that was centrifuged at 10,000 *g*, 20 min, 4 °C to recover the soluble proteins in the supernatant, and a second tube (10 g) to determine the total protein content in the sample at pHx. The protein solubility experiments were performed with three independent defatted protein extracts recovered from rapeseed oilseeds (Fig. 1). The total nitrogen contents in the sample at pHx and in the corresponding supernatant at pHx were determined using the Kjeldhal method. The nitrogen-to-protein conversion factor of 5.7 was used (Ntone et al., 2021). The protein solubility (PS, %) was calculated as the ratio between the protein content in the supernatant at a specific pHx and the total protein content in the sample at pHx. The PS formula is as follows:

$$\text{PS}\left(\%\right)=\frac{\text{protein}\;\text{content}\;\text{in}\;\text{the}\;\text{supernatant}\;\text{at}\;a\;\text{specific}\;\text{pHx}\;}{\text{total}\;\text{protein}\;\text{content}\;\text{of}\;\text{the}\;\text{whole}\;\text{sample}\;\text{at}\;\text{pHx}}\;x\;100$$

### 2.9 Statistical analysis

All experiments were carried out at least in triplicate; the results were expressed as mean values ± standard deviation. The General Linear Model procedure of Statgraphics Plus, version 5 (Statistical Graphics Corp., Englewood Cliffs, NJ, USA) was used to perform analyses of variance (ANOVA). Differences between the treatment means were compared at the 5 % level of significance using Fisher's least significance difference (LSD) test.

## 3 Results and discussion

### 3.1 Microstructure of the aqueous extracts recovered from rapeseed oilseeds

The integrated green biorefinery process used in this study was based on previous studies dedicated to aqueous extractions from various oilseeds (hemp seeds, chia seeds, camelina seeds) and walnut kernels (Lopez et al., 2021, 2023a, 2023b) and adapted to rapeseed oilseeds (Fig. 1). Indeed, in the previous studies, the oilseeds were soaked and grinded in water as aqueous media. In the case of rapeseed oilseeds, the aqueous extraction performed with water induced the release of materials with a decrease in pH down to pH 5.6 and the recovery of a floculated aqueous extract as observed at the macroscopic level. CLSM observations revealed the formation of aggregates of rapeseed OBs and OBs of large size formed by coalescence (Fig. 2 A-1). This result was in agreement with the aqueous extraction from rapeseed oilseeds performed with water reported in literature (Romero-Guzmán et al., 2020c; De Chirico et al., 2018). The aqueous extraction performed in alkali conditions at pH 9.5 allowed the release and the recovery of individual oil droplets that corresponded to the natural rapeseed OBs (Fig. 2 A-2**). This was in agreement with previous studies reporting the recovery of rapeseed OBs using bicarbonate-based soaking and grinding media at pH 9.5** (De Chirico et al., 2018)**. The CLSM images showed the dispersion of OBs and the presence of proteins in the surrounding aqueous phase (**Fig. 2 A-2). The use of a buffer with a high alkalinity (pH 9.5) for soaking and grinding avoided aggregation and coalescence of rapeseed OBs, probably due to repulsion forces between OBs and to the high solubility of rapeseed proteins at alkali pH. The alkali aqueous process used in this study permitted the co-extraction of rapeseed OBs and proteins from rapeseed oilseeds. In order to further understand the impact of experimental conditions on the extraction of rapeseed OBs, the role of salts such as NaCl was investigated. The aqueous extraction performed at pH 9.5 in presence of 100 mM NaCl showed aggregates of rapeseed OBs (Figs. 2 A-3). The impact of monovalent ions such as Na^+^ from NaCl on the physical instability of OBs extracted from various oilseeds and walnuts has been already reported and discussed (Lopez et al., 2021, 2023b; Zhou et al., 2022). NaCl induced electrostatic screening at the surface of OBs and weakened the electrostatic repulsions between OBs promoting their aggregation. We concluded that NaCl had a negative impact on the microstructure of the alkali aqueous extract recovered from rapeseed oilseeds compared to the extraction performed at pH 9.5 in absence of NaCl.

**Fig. 2:**
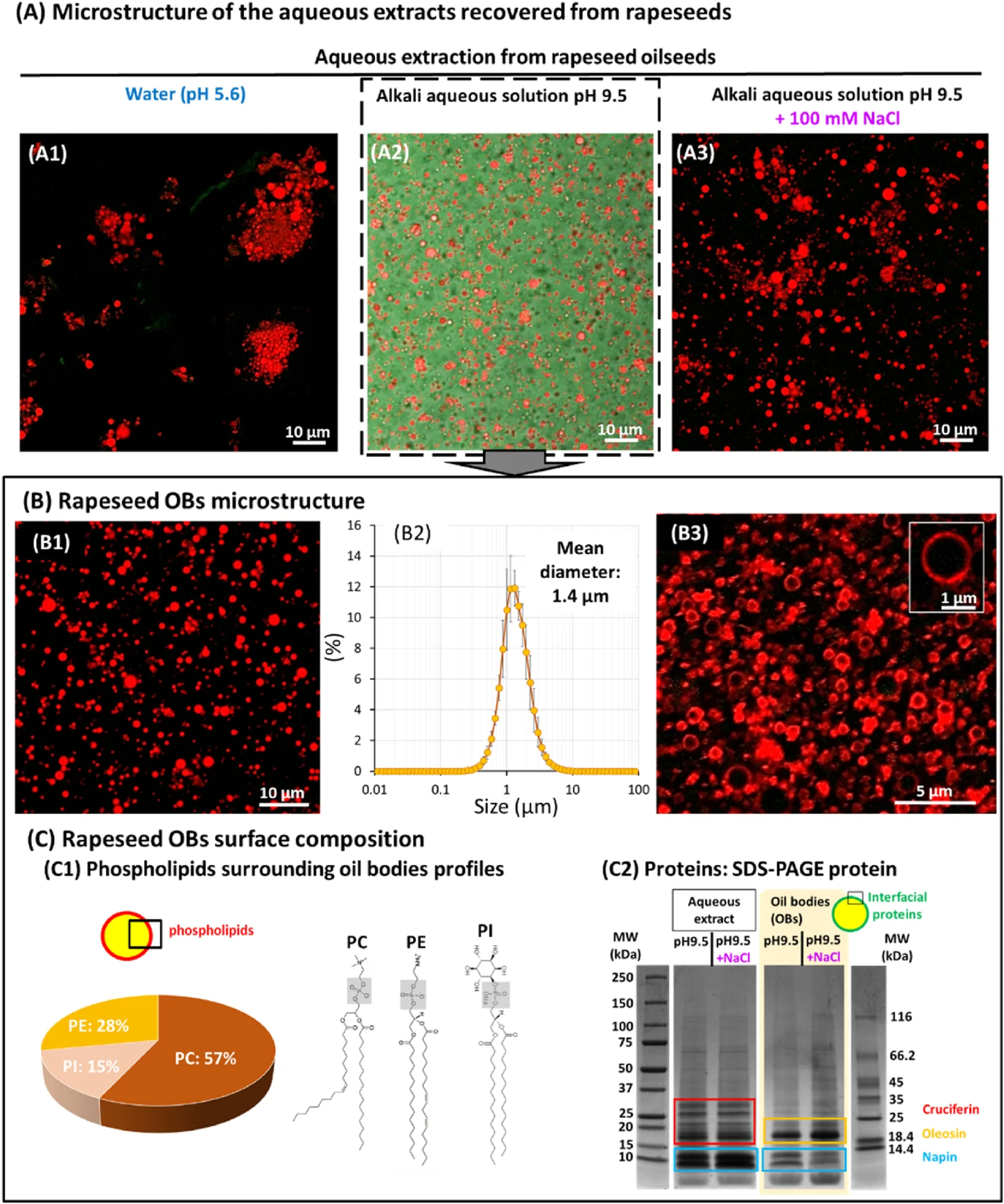
Aqueous extracts recovered from rapeseeds with a focus on the microstructure and surface composition of oil bodies (OBs). **(A)** Microstructure of the aqueous extracts recovered with water (A1), with an alkali aqueous solution at pH 9.5 in absence (A2) or presence of 100 mM NaCl (A3) observed by confocal laser scanning microscopy (CLSM) alone (A1 and A3) or combined with DIC. The neutral lipids and proteins are labelled with nile red (red colour) and fast green (green colour) fluorescent dyes, respectively. **(B)** Microstructure of rapeseed OBs examined by CLSM showing the internal neutral lipid core labelled with nile red fluorescent dye (B1) or the interfacial layer labelled with Rhodamine-DOPE fluorescent dye (B3) and particle size distribution of the rapeseed OBs dispersed in the alkali aqueous extract (B2). **(C)** Surface composition of the OBs including the phospholipid composition determined by HPLC (C1) and the protein composition determined by SDS-PAGE in reducing conditions.

This study showed that the pH and salt environment upon aqueous extraction affected the surface properties of rapeseed OBs with consequences on their physical stability, as previously highlighted (Romero-Guzmán et al., 2020c; De Chirico et al., 2018). Also, this study showed that the behaviour of rapeseed OBs was different from the behaviour of other sources of OBs upon aqueous extraction. The biodiversity of the oilseeds and the specificity of the OB surface composition including the presence of exogenous storage proteins require the adaptation of the aqueous extraction process, i.e. at least the pH and ionic strength.

This green refinery process avoid solvent use and preserve the integrity of OBs but must be balanced with potential challenges, such as the consumption of water used for the aqueous extraction.

### 3.2 Rapeseed OBs recovered by alkali aqueous extraction

#### 3.2.1 Microstructure and surface composition

CLSM images with nile red as fluorescent dye to visualize the neutral lipids (i.e., the TAGs) showed the OBs recovered by alkali aqueous extraction from rapeseed oilseeds (Fig. 2-B1). The extraction performed at pH 9.5 (buffer in absence of salt) favoured the recovery of individual spherical OBs that were well-dispersed in the volume of the aqueous extract. The aqueous extract recovered from rapeseed oilseeds is therefore a natural oil-in-water emulsion in which the TAGs are entrapped in spherical oil droplets corresponding to OBs.

The size distribution of the rapeseed OBs recovered by alkali aqueous extraction spanned from 0.2 to 10 μm (Fig. 2-B2). Their mean diameter was 1.4 ± 0.1 μm and their modal diameter was 1.2 ± 0.2 μm (n = 6 independent alkali aqueous extractions from rapeseed oilseeds). These results are in agreement with scientific literature. Diameters below 2 μm were observed from transmission electron microscopy images for rapeseed OBs within the seeds (Boulard et al., 2015). The size distributions and the diameters of rapeseed OBs were in agreement with literature for aqueous extraction at alkali pH 9.5 (Romero-Guzmán et al., 2020c; De Chirico et al., 2018).

The CLSM images obtained with the Rh-DOPE fluorescent dye showed a continuous ring around each rapeseed OB, corresponding to the presence of a layer of phospholipids that delimit the hydrophobic core of TAGs (Fig. 2, Fig. 3). This is in agreement with the presence of a monolayer of phospholipids at the surface of OBs (Nikiforidis, 2019b). Around rapeseed OBs, the layer of phospholipids appeared homogeneous. This layer of phospholipids envelopping OBs was previously observed by CLSM for hemp seed OBs (Lopez et al., 2021), chia seed OBs (Lopez et al., 2023a), camelina seed OBs (Lopez et al., 2023a), walnut kernel OBs (Lopez et al., 2023b), coconut OBs (Dave et al., 2019) and almond OBs (Gallier et al., 2012).

The surface composition of the rapeseed OBs recovered by alkali aqueous extraction was analyzed.

The phospholipid content was 270 mg per 100 g of lipids (0.27 wt% of the total lipid content). Phospholipids are amphiphilic molecules characterized by a hydrophilic head group and a hydrophobic hydrocarbon tail. They play key functional roles at the surface of rapeseed OBs where they form a monolayer embedding OB endogenous proteins (oleosins, caleosins, steroleosins) and encapsulating the TAG core (Nikiforidis, 2019b). The analysis of the phospholipid fraction revealed the presence of 3 main species: phosphatidylcholine (PC), phosphatidylinositol (PI) and phosphatidylethanolamine (PE) that accounted for 57 wt%, 15 wt% and 28 wt% of total phospholipids respectively (Fig. 2-C1), in agreement with literature (Tzen et al., 1993). The zwitterionic PC and the anionic PI that imparts a negative charge for pH 7.0 and above are sensitive to changes in pH values (amount of H^+^ ions) and in their ionic environment due to salts such as monovalent ions (e.g. NaCl) and divalent ions (e.g., CaCl_2_).

The proteins present in the alkali aqueous extracts and at the surface of the rapeseed OBs were identified by SDS-PAGE in reducing conditions (Fig. 2-C2). In the whole aqueous extracts, the main proteins corresponded to cruciferin (several bands with MW above 18 kDa) and to napin (2 bands below 15 kDa). This in in agreement with the protein composition of rapeseeds (Wanasundara et al., 2016b). At the surface of the rapeseed OBs, the proteins corresponded to the main endogenous integral proteins of OBs, i.e. the oleosin isoforms (intense bands 15-20 kDa). The analysis of the proteins located at the surface of OBs revealed that exogenous seed proteins were also present at the surface of the rapeseed OBs recovered during the process of alkali aqueous extraction. These exogenous proteins were the napin (2 intense bands below 15 kDa), and the cruciferin (bands between 20 and 35 kDa) but in low amount according to the density of the bands (Fig. 2-C2). These results revealed the preferential adsorption of napin compared to cruciferin at the surface of the rapeseed OBs recovered by alkali aqueous extraction. The rapeseed OBs recovered by alkali aqueous extraction in presence of 100 mM NaCl contained oleosins and napins at their surface. The analysis of the relative density of the bands could be interpreted as a higher amount of oleosins than napins after extraction with NaCl compared to without NaCl (Fig. 2-C2). We showed that rapeseed OBs were recovered in the alkali aqueous media together with co-extracted materials, i.e. exogenous seed proteins. Interactions between OBs and co-extracted proteins were evidenced by SDS-PAGE. The high alkalinity buffer used in this study did not disrupt the interactions (i.e., ionic bonds, hydrophobic interactions) that connected exogenous proteins (i.e. napins) with the surface of OBs. Our results are in agreement with literature. Other authors reported the presence of oleosins and napins at the surface of OBs extracted at pH 9.5 with NaHCO_3_ (0.1 mol/L) using a blender for grinding (Romero-Guzmán et al., 2020c). It has been previously reported that when protein mixtures with OBs are extracted, storage proteins are forming a film around the OBs by interacting with the OBs membrane proteins through hydrophobic and van der Waals attractive forces (Ntone et al., 2021). The amount of exogenous proteins adsorbed at the surface of rapeseed OBs depends on the grinding medium and to washing steps as already discussed in literature (De Chirico et al., 2018).

#### 3.2.2 Lipid composition of rapeseed OBs recovered by alkali aqueous extraction

A detailed analysis of the lipid composition of the OBs recovered by alkali aqueous extraction from rapeseed oilseeds was performed.

##### 3.2.2.1 Fatty acid composition

The fatty acid (FA) composition of rapeseed OBs presented Fig. 3-A revealed that three FA accounted for more than 5 wt% of the total FA: the oleic acid (18:1 *n-*9, O: 62.3 wt%), the linoleic acid (18:2 *n-*6, LA: 19.9 wt%) and the α-linolenic acid (18:3 *n-*3, ALA: 8.5 wt%). Both.

**Fig. 3:**
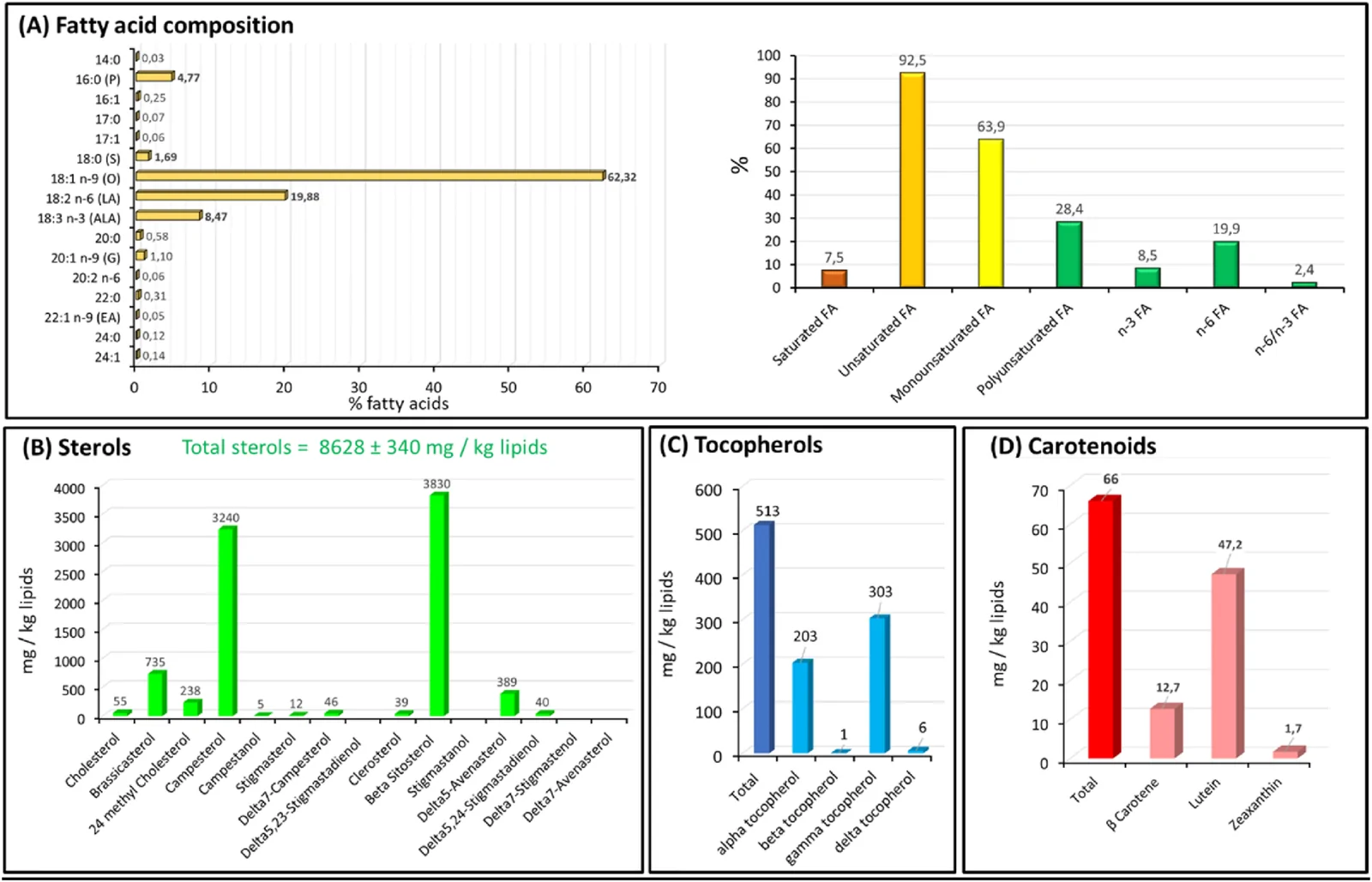
Lipid composition of rapeseed oil bodies (OBs) recovered by alkali aqueous extraction. **(A)** Fatty acid composition with individual fatty acids (left) and sums of fatty acids (right). **(B)** Sterol composition, **(C)** tocopherol composition, **(D)** carotenoid composition.

LA and ALA are essential FA for humans since they cannot be synthesized in the human body and must be consumed from the diet. The unsaturated FA corresponded to 92.5 wt% of the total FA in the OBs with 63.9 wt% of monounsaturated FA and 28.4 wt% of polyunsaturated FA among them 19.9 wt% of *n-*6 FA and 8.5 wt% of *n-*3 FA. Rapeseed OBs also contained saturated FA, mainly the palmitic acid (16:0, P: 4.8 wt%) and the stearic acid (18:0, S: 1.7 wt%). The erucic acid (22:1 *n-*9) accounted for 0.05 wt% of total FA, which is low and in accordance with human consumption of rapeseed OBs. The high content in unsaturated FA, especially the polyunsaturated FA, and the *n-*6/*n-*3 FA ratio of 2.4 make rapeseed OBs of high nutritional value and provides health benefits as already reported for rapeseed oil (Shen et al., 2023).

##### 3.2.2.2 Unsaponifiable fraction: plant sterols, tocopherols and carotenoids

The unsaponifiable fraction of rapeseed OBs contained plant sterols (Fig. 3-B), tocopherols (Fig. 3-C) and carotenoids (Fig. 3-D). These intrinsic phytochemicals showed that rapeseed OBs are natural reservoirs and vectors of bioactive components that are liposoluble.

**Plant sterols.** The plant sterols quantified in the rapeseed OBs recovered by alkali aqueous extraction accounted for 8628 ± 340 mg/kg of lipids. The amounts of the individual species of plant sterols are presented Fig. 3-B. The main plant sterols were the β-sitosterol (3830 ± 165 mg/kg; 44.2 wt% of total sterols), the campesterol (3240 ± 131 mg/kg; 37.4 wt% of total sterols) and the brassicasterol (735 ± 25 mg/kg; 8.5 wt% of total sterols). These amounts of plant sterols are in agreement with values reported for rapeseed oil (Shen et al., 2023). Plant sterols are particularly known for their cholesterol-lowering properties in adults (Zhang et al., 2022). The use of plant OBs from oilseeds in food formulations is therefore gaining interest since they could contribute to reaching the daily intake of plant sterols (2g/day) that are required for the dietary prevention of cardiovascular diseases in adults. However, the formulation of rapeseed OBs in foods for infants including follow-on formula should be further evaluated as regards to the competition between plant sterols and cholesterol naturally provided in human milk and the potential long-term consequences, as previously highlighted (Lopez et al., 2023b).

**Tocopherols.** The total amount of tocopherols recovered in the rapeseed OBs was 513 ± 27 mg/kg of lipids. The main tocopherols were the γ-tocopherol (303 ± 14 mg/kg; 59.1 wt% of total tocopherols) and the α-tocopherol (203 ± 13 mg/kg; 39.6 wt% of total tocopherols) (Fig. 3-C). Tocopherols that are known to be very efficient natural antioxidants are intrinsically associated to rapeseed OBs and their antioxidative activity is involved in the oxidative stability of rapeseed OBs.

**Carotenoids.** The amount of carotenoids in the rapeseed OBs was 66.0 ± 2.4 mg/kg, mainly the lutein (47.2 ± 1.2 mg/kg; 71.6 wt% of carotenoids) that is important for healthy eye function, and the β-carotene (12.7 ± 0.8 mg/kg; 19.3 wt% of total carotenoids) (Fig. 3-D). These results are in agreement with the contents in carotenoids in non refined rapeseed oil although discrepancies exist between studies (Shen et al., 2023).

As a conclusion, this analytical study showed the nutritional and health interests of rapeseed OBs that contain unsaturated FA species with a *n-*6/*n-*3 FA ratio of 2.4, and also biologically active compounds such as plant sterols, tocopherols, carotenoids and phopholipids (Fig. 2 C1). The high amount of polyunsaturated FA of health interest is naturally protected by the natural structure of rapeseed OBs that carry intrinsic antioxidants able to resist lipid oxidation.

#### 3.2.3 Impact of pH on the physical stability of rapeseed OBs

The physical stability of dispersions of rapeseed OBs was investigated as a function of pH with a multiscale approach. The results will contribute in a better understanding of their physical behaviour in food formulations which can have a range of pH values including acidic pH (e.g. pH 4 for mayonnaise).

Fig. 4 shows the samples at a macroscopic level (physical stability of the emulsions containing the OBs as a function of pH; Fig. 4-A), at a microscopic scale (microscopy observations; Fig. 4-B) and changes in the zeta potential value of rapeseed OBs as a function of pH (Fig. 4-C).

**Fig. 4:**
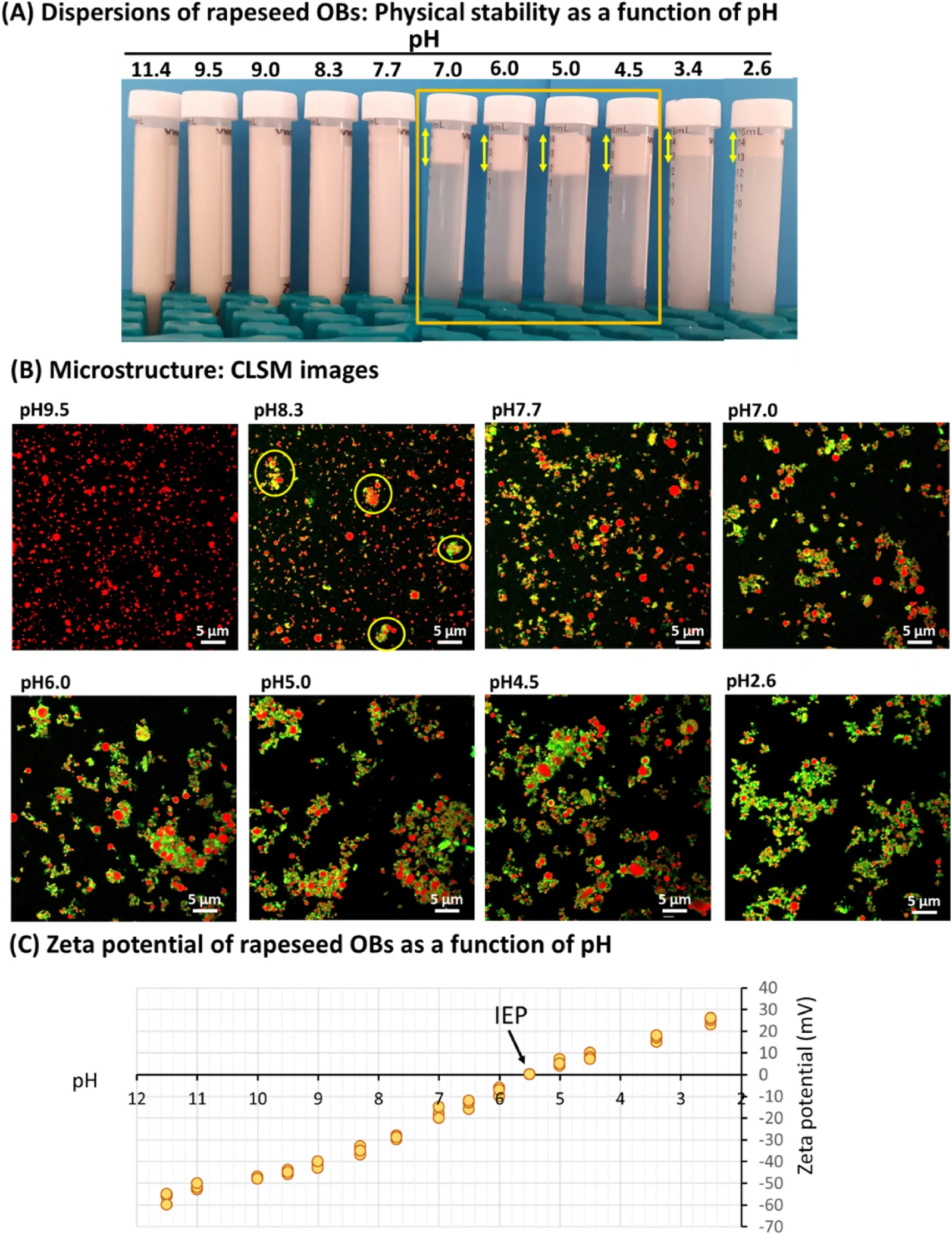
Behaviour of rapeseed oil bodies (OBs) as a function of pH. **(A)** physical stability of dispersions of rapeseed OBs as a function of pH. **(B)** Confocal laser scanning microscopy images taken as a function of pH with Nile red (red colour) to label the internal lipid core and fast green FCF to label the proteins (green colour). **(C)** Changes in the zeta potential of rapeseed OBs as a function of pH; IEP: isoelectric point.

At the macroscopic level, the samples appeared homogeneously distributed in the volume of the tubes from pH 11.4 to pH 7.7. For pH 7.0 and below, a phase separation appeared in some tubes within 2 h of storage, with no further evolution after 5 days of storage. This phase separation was interpreted as a physical destabilisation of the dispersions of rapeseed OBs with the formation of a OB-rich layer at the top of the tubes. Between pH 7.0 and pH 4.5, the bottom of the tubes appeared clear. For pH 3.4 and 2.6, the OB-rich cream layer was reduced and the turbidity of the bottom of the tubes was increased compared to the pH range from 7.0 to 4.5 (Fig. 4-A). The physical instability of the rapeseed OBs emulsions was observed at a higher pH value (i.e. pH 7.0) than that of chia seed OBs (pH 6.4), camelina seed OBs (pH 6.3), hemp seed OBs (pH 5.8) and walnut kernel OBs (pH 5.5) (Lopez et al., 2021, 2023a, 2023b). This could be attributed to the specific surface composition of rapeseed OBs recovered by alkali aqueous extraction (Fig. 1, Fig. 2).

CLSM observations with the double labelling of neutral lipids and proteins revealed the formation of aggregates of OBs for pH 8.3 and below (Fig. 4-B). The high fluorescence intensity of the fast green dye was attributed to a high density of proteins at the surface of the OBs due to their amount or aggregated state. In the aggregates, the OBs were connected by their protein-rich surface. The size of the OB-rich aggregates increased as a function of the decrease in pH. The large size of the aggregates of OBs associated to the lower density of lipid-rich OBs in comparison to water induced the formation of the OBs-rich cream layer at the top of the tubes observed at the macroscopic level for pH 7.0 and below (Fig. 4-A).

The zeta potential of rapeseed OBs investigated as a function of pH showed changes from −60 mV at pH 11.4 to +25 mV at pH 2.6 with an IEP characterized at pH 5.5 (Fig. 4-C). At pH 7.0, for which phase separation was observed in the tubes (Fig. 4A) and OB aggregates were characterized by CLSM (Fig. 4B), the zeta potential value was – 17.7 ± 2.5 mV. The aggregation of OBs could be at least partially due to low zeta potential values and then to low electrostatic repulsions between OBs for pH 7.0 and below. Aggregation of OBs was previously reported for zeta potential values below −20 mV (Lopez et al., 2021, 2023a, 2023b).

### 3.3 Rapeseed proteins recovered by alkali aqueous extraction

#### 3.3.1 Impact of pH on the solubility of rapeseed protein extracts

The solubility is often considered to be a prerequisite for the performance of proteins in food applications since poor solubility is related to poor technological functionalities. It is also well-accepted that the solubility and physical stability of protein extracts are key parameters involved in the stabilization of oil droplets in O/W emulsions and the overall physical stability of the emulsions. The impact of pH on the physical stability and solubility of the defatted protein aqueous extracts recovered from rapeseeds (Fig. 1) was therefore investigated (Fig. 5). The defatted rapeseed protein extracts (protein content 1 wt%) were adjusted to various pH values. Changes in pH induced changes in the colour of the defatted protein aqueous extracts from yellowish at acidic pH to dark brown above pH 7.0 (Fig. 5 A). This was due to the presence of phenolic compounds which form quinones after oxidation in alkaline conditions that can react with proteins resulting in a brown colour in the protein dispersions (Xu and Diosady, 2002).

**Fig. 5:**
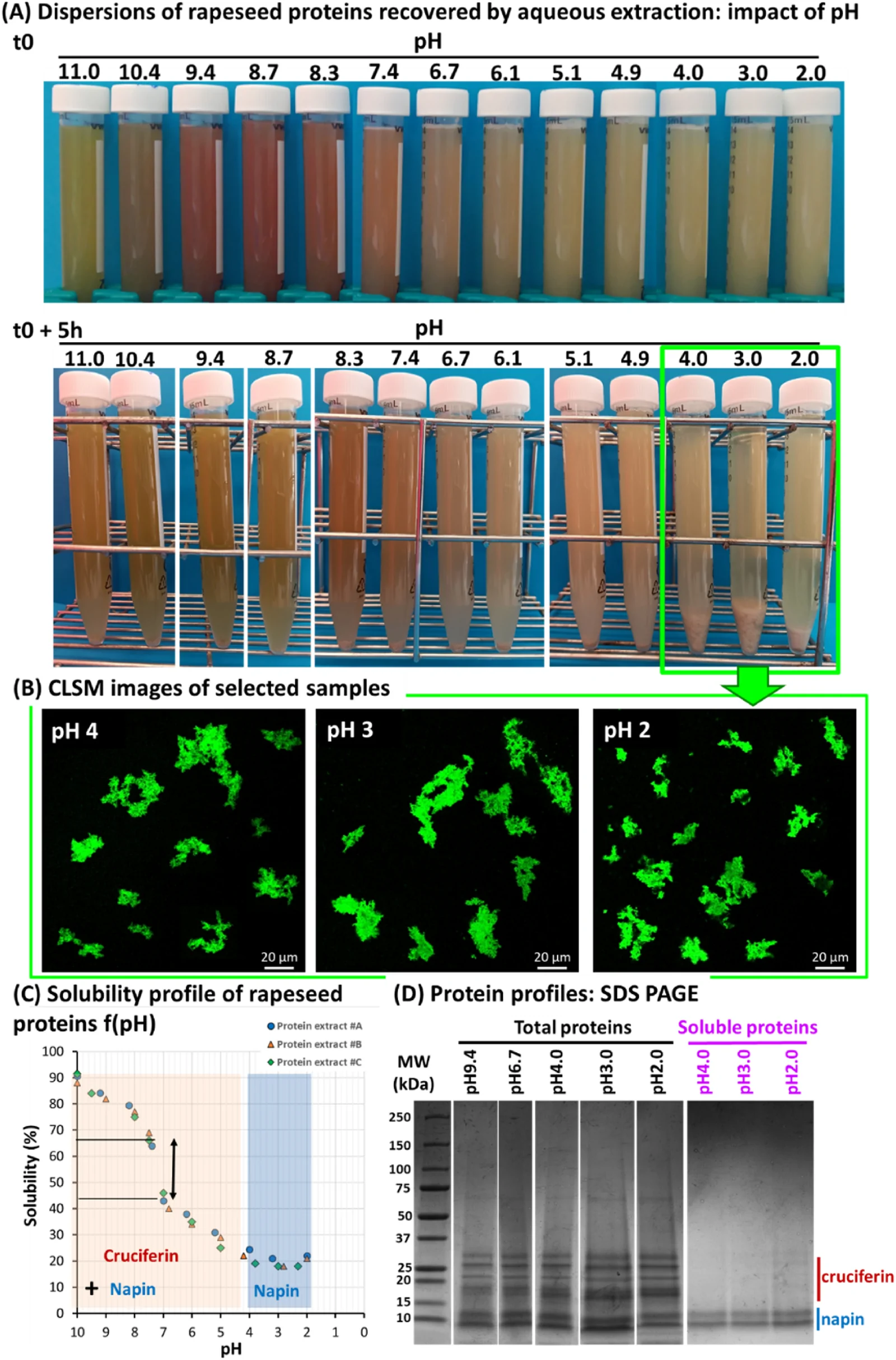
Dispersion of rapeseed proteins recovered by alkali aqueous extraction. **(A)** Photos showing the colour and physical stability of defatted rapeseed protein extracts as a function of pH. **(B)** Confocal laser scanning microscopy images showing proteins aggregates labelled with fast green FCF fluorescent dye. **(C)** Solubility of rapeseed proteins as a function of pH. **(D)** Protein profiles determined by SDS-PAGE.

The physical stability of the defatted rapeseed protein aqueous extracts at various pH values was observed at the macroscopic level after 5 h storage at 4 °C (Fig. 5 A). For pH values in the range from 11.0 to around 5.0, the rapeseed protein dispersions appeared homogeneous and did not reveal any phase separation. For pH values of pH 4.0 and below, the rapeseed protein dispersions spontaneously sedimented at the bottom of the tubes (Fig. 5 A). CLSM observations of the rapeseed protein dispersions stabilized at pH 4.0, pH 3.0 and pH 2.0 revealed the formation of aggregates of proteins at the microscopic level as shown in the CLSM images (Fig. 5 B).

The solubility profile of the rapeseed proteins contained in the defatted protein extract was investigated as a function of pH and is presented Fig. 5C. In this study, the solubility of rapeseed proteins recovered by alkali aqueous extraction was defined as the amount of soluble proteins in the supernatant of a dispersion after centrifugation under defined conditions, as previously performed for camelina seed proteins (Lopez et al., 2024). The solubility of the rapeseed proteins evolved as a function of pH, with the highest solubility at alkaline pH (pH 10.0 – 8.0; more than 75 % solubility) and the lowest solubility between pH 4.0 and 2.0 (about 20 % solubility). A drop in the solubility was observed between pH 7.5 (66.3 ± 2.5 % solubility of the proteins) and pH 7.0 (43.0 ± 2.9 % solubility of the proteins) as highlighted in Fig. 5C. These results are in agreement with the solubility profiles of rapeseed proteins extracted from meals (Gerzhova et al., 2015; Fetzer et al., 2019).

SDS-PAGE protein profiles of the defatted protein extracts showed the presence of both cruciferin and napin whatever the pH (Fig. 5 D) as in the whole aqueous extracts recovered at pH 9.5 (Fig. 2). Both cruciferin and napin exhibited good solubility properties in alkaline media. For acidic pHs, the SDS-PAGE protein profiles revealed that the soluble proteins corresponded only to napin (Fig. 5 D). We interpreted that cruciferin proteins were not soluble for acidic pH values, that they aggregated as revealed by CLSM (Fig. 5 B), and sedimented at the bottom of the tubes (Fig. 5 A) due to the higher density of the aggregated cruciferins than the density of water. The drop of solubility between pH 7.5 and pH 7.0 and the low solubility of rapeseed proteins reported for acidic pHs were ascribed to the aggregation of cruciferin while napin remained soluble. Similar decrease in the solubility of the proteins, mixture of cruciferin and napin, was observed for camelina seed protein aqueous extracts as a function of pH (Lopez et al., 2024). The results found for rapeseed proteins recovered by alkali aqueous extraction are in agreement with literature since a recent study revealed that the insoluble cruciferin fraction formed aggregates at pH ≤ 7, with a partial dissociation of the cruciferin hexamer at pH 6 and below (Mudau et al., 2024). According to literature, cruciferin is no longer significantly negatively charged below its isolectric point around 7.2 which contributes to its aggregation, can undergo dissociation and denaturation at an acidic pH that lead to precipitation of cruciferin aggregates, while napin shows good solubility across the full pH spectrum since its IEP is between 10.0 and 11.0 (Fetzer et al., 2020). In rapeseed protein dispersions containing both cruciferin and napin, cruciferin aggregates formed at pH ≤ 7 do not strongly interact with napin, and contain therefore only cruciferin leaving the napin in solution (Mudau et al., 2024). These pH-dependent properties of rapeseed proteins are used in the acidic precipitation of cruciferin performed at pH 4.0 for its specific recovering from defatted rapeseed meal while napin remains in the soluble supernatant (Fetzer et al., 2020; Akbari and Wu, 2015).

#### 3.3.2 Potentialities of rapeseed proteins recovered by aqueous extraction to stabilize oil-in-water emulsions

The defatted protein extract recovered from rapeseed oilseeds (protein content 1 wt%) was adjusted to pH 9.5 (pH of extraction) or to pH 7.5. The objective was to determine the impact of alkali pH (pH 9.5, 85.1 ± 1.2 % solubility of the proteins Fig. 5 C) or pH close to neutrality but with a high solubility of the whole proteins (pH 7.5, 66.3 ± 2.5 % solubility of the proteins Fig. 5 C) on the capacity of rapeseed proteins to stabilize O/W emulsions. Since i) the solubility of cruciferin is decreased for pH ≤ 7 with formation of aggregates (Fig. 5 C) (Mudau et al., 2024), and ii) aggregates of rapeseed protein coated oil droplets were previously observed at pH 7.0 (Ntone et al., 2021), we decided to investigate higher pH values than pH 7.0. Emulsions were prepared with rapeseed oil to reach a lipid content of 3 wt%, and homogenized at 4 MPa. This low homogenization pressure was chosen in order to prepare large oil droplets and to visualize the oil/water interface by confocal microscopy.

In the O/W emulsions prepared at pH 9.5 and pH 7.5, the mean diameters of the oil droplets measured by laser light scattering were 2.1 ± 0.1 μm and 2.2 ± 0.1 μm, respectively, and were not significantly different (p > 0.05) (Fig. 6 A1 & B1**). The particle size measurements performed without or in presence of SDS (noted –SDS or + SDS, respectively) did not reveal difference (**Fig. 6 **A1 & B1**). This indicated that the oil droplets were not aggregated in the emulsions prepared at pH 9.5 and pH 7.5, which was attributed to electrostatic repulsions between the protein-covered oil droplets. CLSM images confirmed the dispersion of the oil droplets and the absence of aggregation in the emulsions prepared at pH 9.5 and pH 7.5 (Fig. 6 A2 & B2). The size of the oil droplets measured by laser light scattering did not evolve and was therefore not significantly significant (p > 0.05) upon storage at 4 °C for 3 weeks (results not shown). We deduced from these size measurements that the rapeseed proteins recovered by aqueous extraction exhibited good emulsifying properties and that the pH, i.e. pH 9.5 vs pH 7.5, did not affect the functional properties of the rapeseed proteins nor the physical stability of the oil droplets upon storage. Authors reported the aggregation of oil droplets in emulsions prepared with rapeseed protein extracts at pH 7.0 (Ntone et al., 2021). We deduced that the aggregation of the oil droplets can be avoided for pH values above pH 7.0 and that the physical stability of the emulsion depends on the pH.

**Fig. 6:**
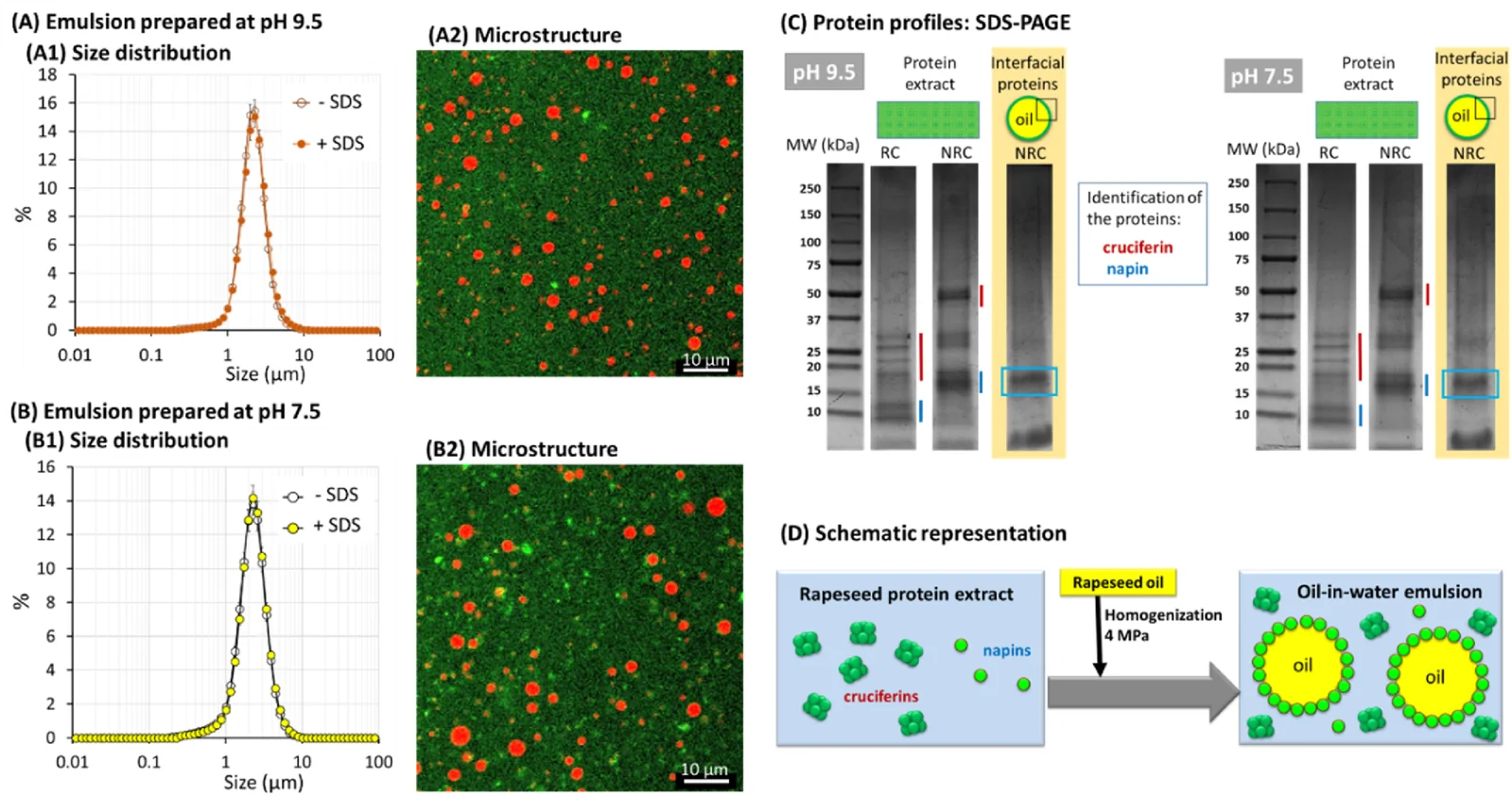
Microstructure and surface composition of emulsions prepared with rapeseed protein extracts. **(A)** Emulsions prepared at pH 9.5 with (A1) characterization of the oil droplet size distribution and (A2) confocal laser scanning microscopy (CLSM) images showing the internal oil core labelled with nile red fluorescent dye and proteins labelled with fast green FCF. **(B)** Emulsions prepared at pH 7.5 with (B1) characterization of the oil droplet size distribution and (B2) CLSM images showing the internal oil core labelled with nile red fluorescent dye and proteins labelled with fast green FCF. **(C)** Protein profiles determined by SDS-PAGE. **(D)** Schematic representation.

The protein profiles in the rapeseed protein dispersions adjusted to pH 9.5 and pH 7.5, and the interfacial protein composition in the emulsions were determined by SDS-PAGE (Fig. 6 C). Whatever the pH, the protein dispersions contained cruciferin (several bands above 18 kDa in reducing conditions, bands at 50 and 27 kDa in non-reducing conditions) and napin (2 bands below 15 kDa in reducing conditions and 1 band at 17 kDa in non-reducing conditions). At the surface of the oil droplets, the band at 17 kDa detected in the SDS-PAGE profiles in non-reducing conditions revealed the presence of napin for both pH 9.5 and pH 7.5. Cruciferin proteins were not present at the surface of the oil droplets as indicated by the absence of band at 50 kDa. The schematic representation of the protein composition in the defatted protein extract and the preferential adsorption of napin at the surface of the oil droplets is proposed Fig. 6 D. These results are in agreement with previous studies reporting the presence of napins at the surface of oil droplets prepared from a mixture of rapeseed napins and cruciferins (Ntone et al., 2021).

Several mechanisms are involved during the emulsification process of plant proteins, such as the diffusion of the proteins to the O/W interface, the surface denaturation of the proteins to align their hydrophilic moieties to the aqueous phase and the hydrophobic moieties to the oil phase, and the steric stabilization provided by the proteins to prevent oil droplet destabilisation. The presence of napin at the surface of the oil droplets was attributed to its solubility, small shape and hydrophobicity, as previously discussed (Ntone et al., 2021). By studying the interfacial behaviour of rapeseed proteins, authors observed that the interfacial properties of napin dominated the monolayer and emulsion characteristics, while the cruciferin possessed lower surface activity due to its larger size and globular conformation at the surfaces (Krause and Schwenke, 2001). Authors reported that napin proteins adsorbed at the surface of oil droplets due to their small molecular size (radius = 1.7 nm (Ntone et al., 2021);) and low mean molecular area (23 nm^2^ (Krause and Schwenke, 2001);), which allowed them to diffuse faster towards the oil and water interface than cruciferin. Also, napin exhibits a low energy barrier for surface adsorption due to its unique Janus-like structure, with two distinct domains of hydrophilic and hydrophobic amino acids on the protein surface. Cruciferin proteins, due to their large size (radius = 4.4 nm), broad distribution of hydrophobic amino acids, and low surface activity, could not adsorb or displace napin from the surface (Ntone et al., 2021). The weak binding of cruciferin as a secondary layer to the napin primary layer was hypothesized and was reported to be potentially important for the emulsion stability (Ntone et al., 2021).

#### 3.3.3 Impact of pH on the physical stability of the oil-in-water emulsions

The physical stability of the O/W emulsions was investigated as a function of pH (Fig. 7). The emulsions at pH 9.5 and 7.5 were physically stable with a homogeneous distribution of the sample in the tubes (Fig. 7 A). The browning of the emulsion at pH 9.5 compared to the emulsion at pH 7.5 was observed and interpreted as the oxidation of polyphenol coumpounds as previously observed for the defatted rapeseed protein extract (Fig. 5 A) and discussed (Xu and Diosady, 2002). For pH 7.0 and below, the emulsions exhibited phase separation at the macroscopic level with a concentration of the samples at the top of the tubes. After 4 h of storage at 4 °C, a displacement towards the bottom of the tube was observed in the sample at pH 2.5 (Fig. 7 A).

**Fig. 7:**
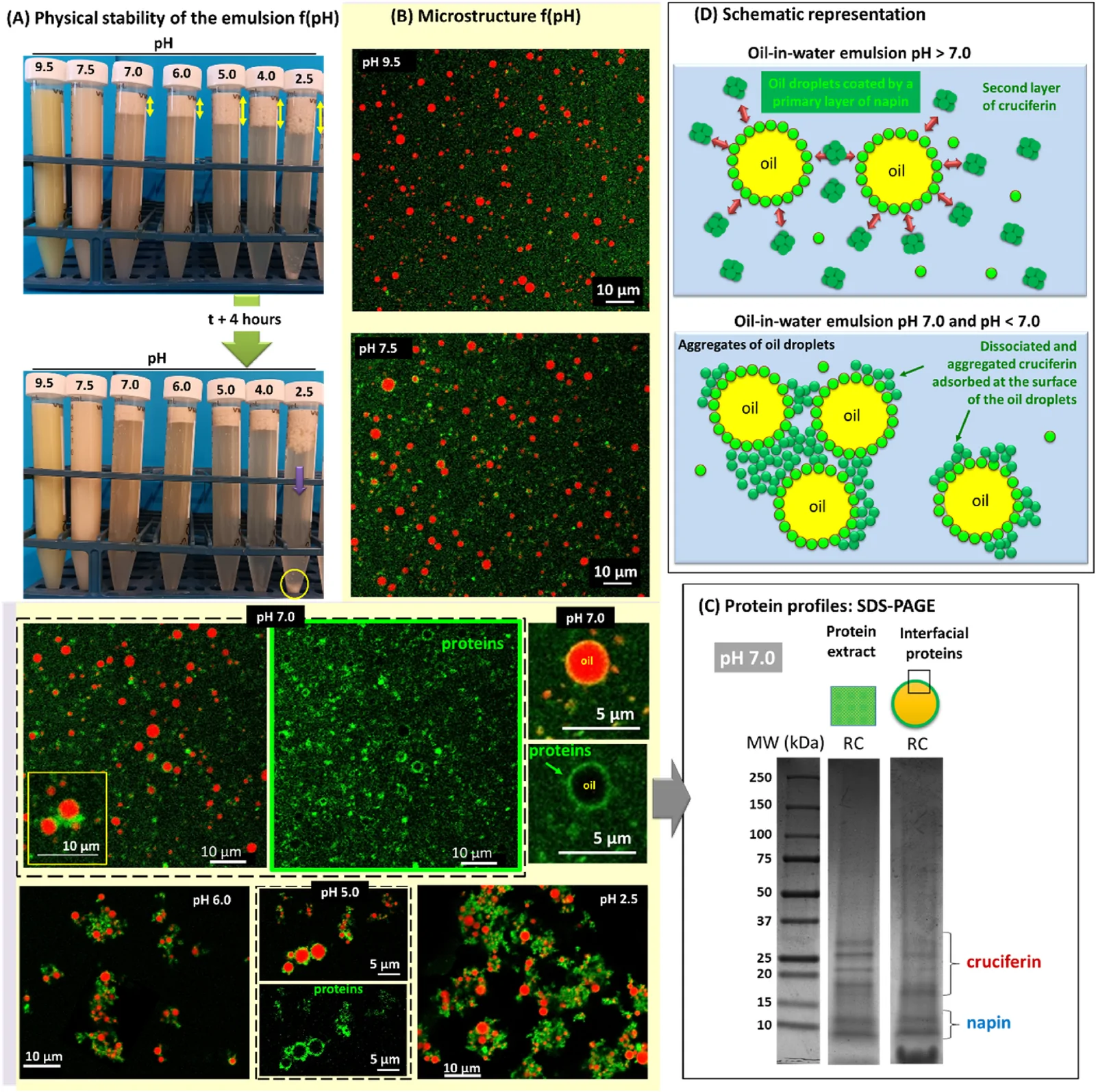
Physical stability as a function of pH of the oil-in-water emulsions prepared with the rapeseed aqueous extracts. **(A)** Photos of the tubes showing the physical stability at the macroscopic scale. **(B)** confocal laser scanning microscopy (CLSM) images showing the internal core of the oil droplets labelled with nile red fluorescent dye (red colour) and proteins labelled with fast green FCF (green colour). **(C)** Protein profiles of the protein extract and interfacial proteins at pH 7.0 determined by SDS-PAGE in reducing conditions (RC). **(D)** Schematic representation.

CLSM images showed the homogeneous distribution of the oil droplets at pH 9.5 and 7.5. In the emulsions at pH 7.0, a dense fluorescent layer was observed around the oil droplets (Fig. 7 B). This was interpreted as a high amount of proteins adsorbed at the surface of the oil droplets, or to the aggregation of proteins at the surface of the oil droplets. Also, the presence of aggregated oil droplets connected by proteins was observed. These microscopic observations revealed the impact of pH on the physical state of the proteins located at the surface of the oil droplets. SDS-PAGE analysis in reducing conditions permitted the characterization of the presence of both napin (2 bands below 15 kDa) and cruciferin (several bands above 18 kDa) at the surface of the oil droplets at pH 7.0 (Fig. 7C). These experimental results provided direct evidence for the presence of cruciferin at the surface of the oil droplets at pH 7.0 whereas cruciferin was absent from the surface of the oil droplets for pH > 7.0 (Fig. 6 C).

For pH < 7.0, the CLSM images revealed the formation of oil droplet aggregates connected by proteins (Fig. 7 B). The creaming of the samples observed at the macroscopic level (Fig. 7 A) was therefore attributed to the oil droplet aggregates that have a lower density than water. In the sample at pH 2.5, the CLSM images revealed the formation of large aggregates composed of oil droplets and proteins (Fig. 7B). The high amount of proteins in these aggregates could be responsible for the increase in the density of the aggregates and to their sedimentation.

The formation of oil droplet aggregates at pH 7.0 and below was unexpected since i) napin was mainly adsorbed at the surface of the oil droplets prepared at pH 9.5 and 7.5 as revealed by SDS-PAGE (Fig. 6 C), ii) napin exibits an IEP between 10.0 and 11.0 ruling out the hypothesis of electrostatic repulsion reduction between napin-coated oil droplets for pH ≤ 7.0, and iii) napin is soluble in the range of pH investigated (Fig. 5 C). However, SDS-PAGE analysis revealed the presence of cruciferin at the surfacce of the oil droplets at pH 7.0 (Fig. 6 C). The drop in the solubility of the rapeseed proteins recovered by aqueous extraction, i.e. 66.3 ± 2.5 % solubility at pH 7.5 and 43.0 ± 2.9 % soubility at pH 7.0 (Fig. 5 C), that is attributed to the denaturation of cruciferin could be involved in the aggregation of the oil droplets occurring for pH 7.0 and below. Although multiple driving forces could be involved, we propose that aggregated cruciferin mediated bridging flocculation could be involved in the physical destabilisation of the emulsion for pH ≤ 7.0. The aggregation of oil droplets at pH 7.0 was previously reported (Ntone et al., 2021), and the weak binding of cruciferin as a secondary layer to the napin primary layer adsorbed at the surface of the oil droplets at pH 7.0 was previously hypothesized (Ntone et al., 2021). At pH 2.5, the high amount of unsoluble and aggregated cruciferin could be responsible for the displacement of the protein-coated oil droplet aggregates towards the bottom of the tube, as observed for the defatted protein suspension below pH 4.0 (Fig. 5 A). A schematic representation of the emulsion as a function of pH highlighting changes in the physical state of cruciferin and its potential key role in the aggregation of the oil droplets is presented Fig. 7 D.

### 3.4 High pressure homogenization of the whole aqueous extracts to tailor food emulsions: impact on rapeseed OBs microstructure and surface composition, and on their physical stability as a function of pH

#### 3.4.1 High pressure homogenized rapeseed OBs

High pressure homogenization is a technological process commonly used in the food industry to decrease the size of the emulsion oil droplets in order to increase the physical stability of the product during its shelf life (Masbernat et al., 2026). The whole rapeseed alkali aqueous extracts recovered from rapeseed oilseeds (Fig. 1), that contained both rapeseed OBs and exogenous plant storage proteins (Fig. 2), were submitted to homogenization under various pressures ranging from 0 to 60 MPa. The increase in the homogenization pressure decreased the mean diameter of the homogenized OBs (noted H-OBs) and increased their specific surface area (Fig. 8 A). The diameter of the natural OBs recovered by aqueous extraction, i.e. 1 0.4 μm (Fig. 2 B2), decreased to 0.2 μm upon homogenization at 60 MPa. The surface of OBs increased from 4.9 m^2^/g oil to 28.3 m^2^/g oil after homogenization at 60 MPa (i.e., 5.8-fold), showing the efficacity of surface-active components to stabilize the surface created under pressure. Similar results were obtained with hemp seed OBs, camelina seed OBs and walnut kernel OBs (Lopez et al., 2021, 2023b, 2024). CLSM images showed the reduction of the size of H-OBs associated to an increase in their number as a function of the increase in the homogenization pressure applied to the whole aqueous extract, and the absence of aggregates (Fig. 8 C). The zeta-potential of the rapeseed OBs was −26.8 ± 1.2 mV and the negative value evolved significantly (p < 0.01) to −30.2 ± 1.3 mV for H-OBs homogenized at 60 MPa (Fig. 8 B). This was related to the changes in surface composition induced by homogenization, and possibly also to structural reorganization at the surface of H-OBs. The negative value of the zeta potential was involved in electrostatic repulsions between H-OBs, avoided their aggregation and contributed in their physical stability.

**Fig. 8:**
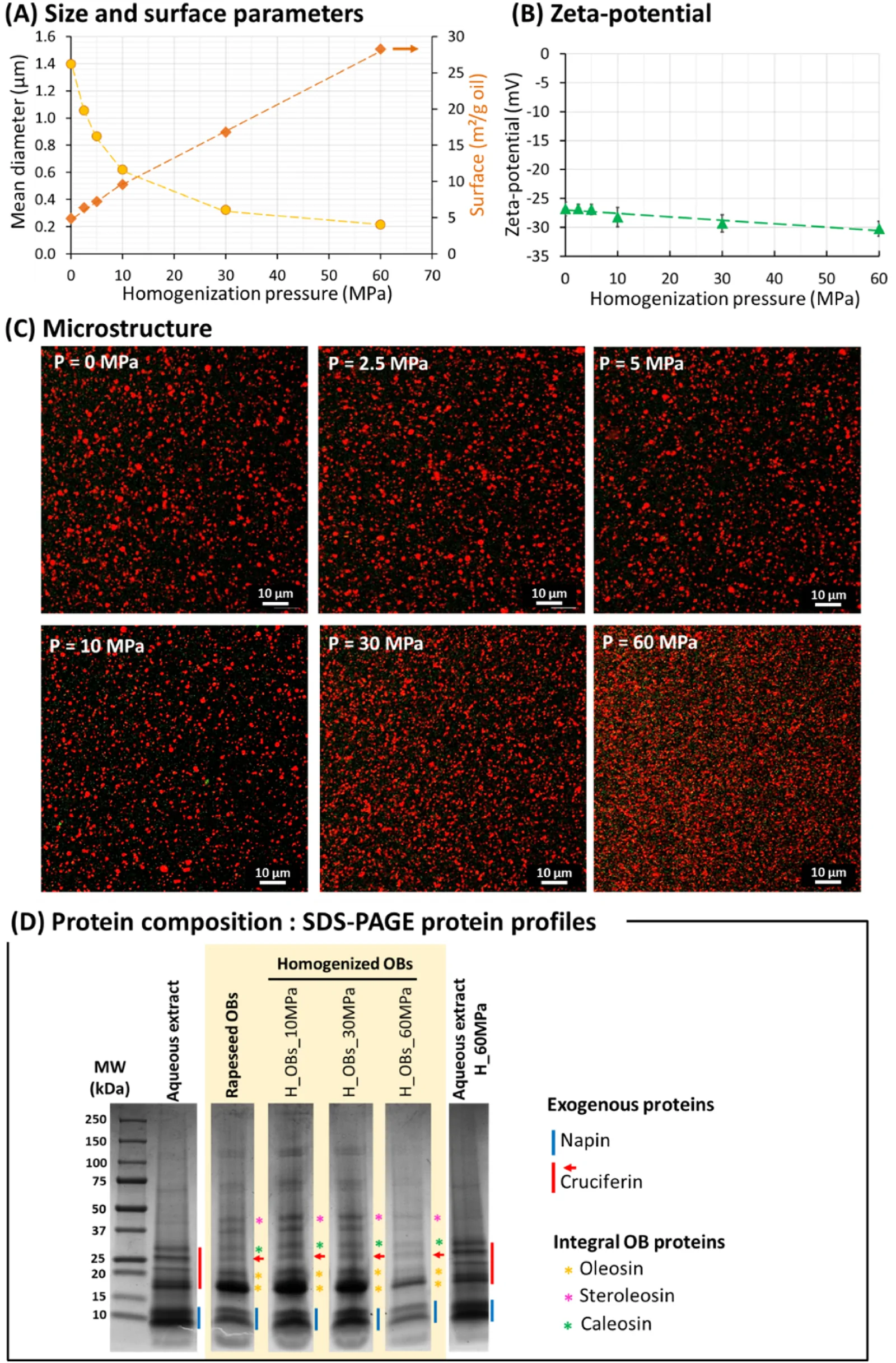
Impact of homogenization on the whole extracts recovered from the alkali aqueous extraction of rapeseed oilseeds. **(A)** Changes in the mean diameter and surface as a function of the homogenization pressure. **(B)** Changes in the zeta potential as a function of the homogenization pressure. **(C)** Confocal laser scanning microscopy (CLSM) images of the whole extracts homogenized as various pressure as indicated. **(D)** Protein composition of the homogenized oil bodies determined by SDS-PAGE in reducing conditions.

The protein profiles of OBs and H-OBs were examined by SDS-PAGE in reducing conditions (Fig. 8 D). The main proteins located at the surface of H-OBs were oleosins (intense bands at 15-20 kDa), caleosins (30 kDa) and napins (2 bands lelow 15 kDa). The band at 27 kDa could be attributed to cruciferin subunits. Cruciferin proteins that were present in the aqueous phase surrounding OBs, did not adsorb massively at the surface of H-OBs. The high cohesivity of the rapeseed OBs could explain the presence of oleosins at the surface of H-OBs homogenized at 60 MPa. The secondary structures of oleosins allow them to remain stable in the phospholipid monolayer on the surface of OBs (Jolivet et al., 2004). This was already discussed for hemp seed H-OBs (Lopez et al., 2021), walnut H-OBs (Lopez et al., 2023b) and camelina seed H-OBs (Lopez et al., 2024). Interestingly, the oleosins present at the surface of rapeseed H-OBs could play a key role, together with the antioxidants (tocopherols) in the protection of PUFAs against oxidation. Indeed, authors reported that oleosins act as a barrier to oxidizing agents such as hydroperoxides and oxygen (Gray et al., 2010).

#### 3.4.2 Impact of pH on homogenized rapeseed OBs

The physical stability of rapeseed H-OBs homogenized at 30 MPa was investigated as a function of pH.

Changes in the pH of the whole rapeseed aqueous extract homogenized at 30 MPa induced changes in the colour (Fig. 9 A). The browning of the samples was observed at alkaline pH values as previously reported for the defatted rapeseed protein extracts (Fig. 5 A) and the emulsions (Fig. 7 A) and attributed to the oxidation of phenolic compounds (Xu and Diosady, 2002). After 4 h storage at room temperature, some samples exhibited phase separation (Fig. 9 B). The samples at pH 7.0 to 5.2 exhibited a layer of materials at the top of the tubes and an aqueous phase at the bottom of the tubes. The samples at pH 4.4 and 3.4 were floculated without any phase separation at the top or bottom of the tubes. The sample at pH 2.5 formed a layer of materials sedimented at the bottom of the tube. During the 14 days of storage at 4 °C, the physical instability of the samples evolved (Fig. 9 C).

**Fig. 9:**
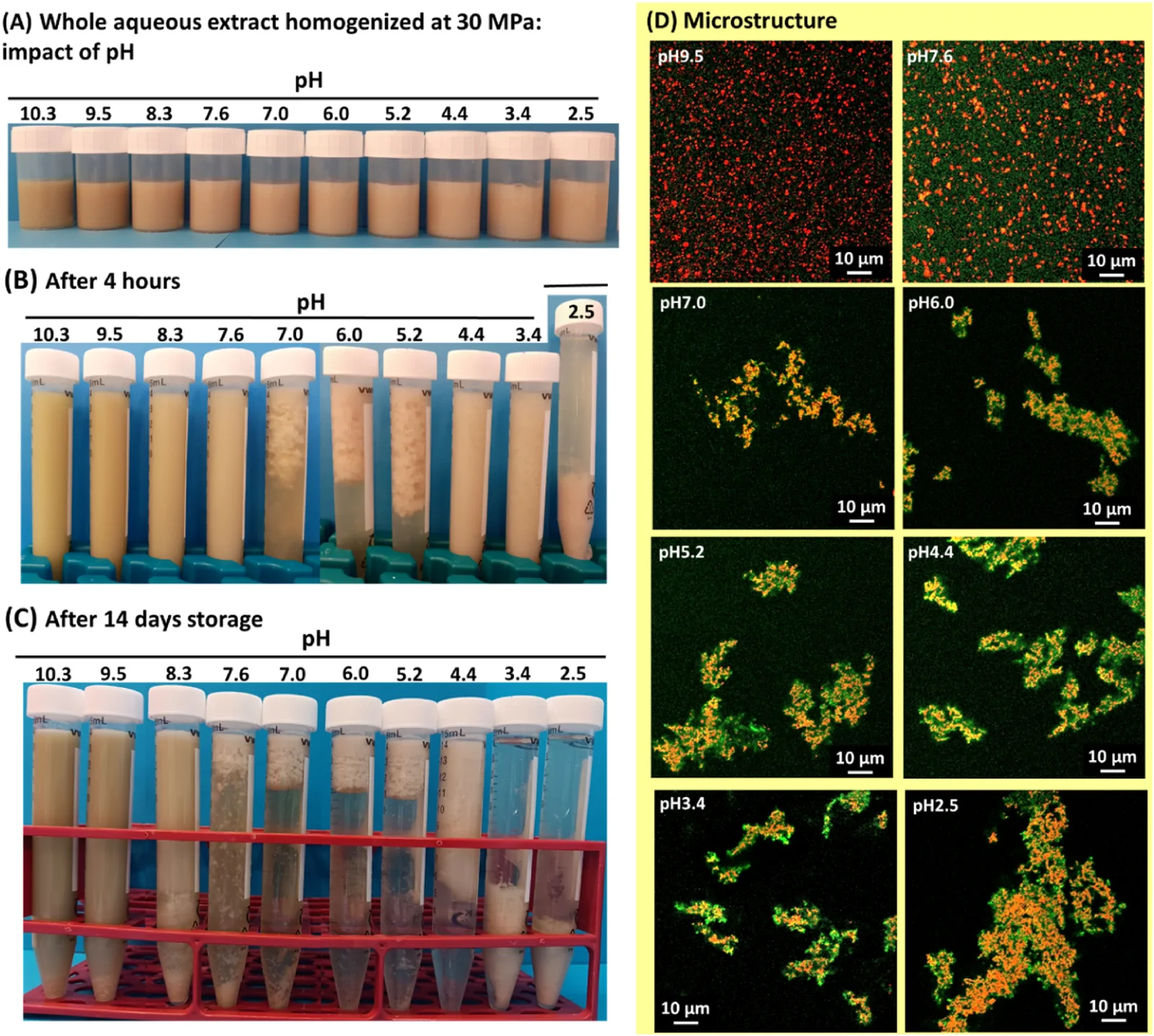
Physical stability as a function of pH of the whole aqueous extract recovered in alkali conditions and homogenized at 30 MPa. The photos of the tubes were taken **(A)** after adjustement in pH, **(B)** after 4 h and **(C)** after 14 days storage. **(D)** Confocal laser scanning microscopy (CLSM) images of the whole extracts homogenized at 30 MPa at given pH values as indicated in the figures. The homogenized oil bodies were labelled with nile red fluorescent dye (red colour) and the proteins were labelled with fast green FCF (green colour).

CLSM images taken after 5 h storage of the samples at 4 °C allowed a better understanding of their physical destabilisation as a function of pH (Fig. 9 D). At pH 9.5, the small H-OBs (mean diameter 0.32 μm) were dispersed in the aqueous extract. At pH 7.6, small aggregates of H-OBs were observed. For pH 6 and below, large aggregates of H-OBs were observed.

As a function of the decrease in pH, additional proteins adsorbed at the surface of the H-OBs or connected with the interfacial proteins to form a kind of network. In the samples at pH values 7.0 to 5.2, the large aggregates were responsible for the layer formed at the top of the tubes. The shift of the aggregates towards the top of the tubes (Fig. 9 A) was interpreted as due to the lower density of the small H-OBs than the surrounding aqueous phase. In the sample at pH 2.5, the sedimentation of the H-OBs aggregates was attributed to the amount and/or kind of proteins adsorbed at the surface of the H-OBs. We interpreted that cruciferin, that is insoluble at this acidic pH (Fig. 5 C), affected the physical stability of the H-OBs as a function of pH and played a key role in the sedimentation of the H-OBs aggregates. The samples at pH 4.4 and 3.4 were a kind a intermediate in the role played by cruciferin in the physical destabilisation of the H-OBs aggregates.

## 4 Conclusions

This study demonstrates that alkali aqueous extraction enables the simultaneous recovery of native rapeseed OBs and storage proteins while preserving their intrinsic nutritional and structural attributes. More importantly, it reveals that the functionality of these aqueous extracts is governed not only by the characteristics of the OBs themselves, but also by the dynamic interactions established with co-extracted proteins throughout processing. Rapeseed OBs were shown to be naturally structured lipid carriers enriched in nutritionally valuable unsaturated fatty acids, phytosterols, tocopherols and carotenoids, making them attractive clean-label ingredients for plant-based food formulations. However, their colloidal behaviour cannot be dissociated from that of the surrounding protein phase. The preferential adsorption of napin at oil/water interfaces, together with the contribution of cruciferin to pH-dependent aggregation phenomena, demonstrates that the composition of the interfacial layer evolves during processing and ultimately determines emulsion stability. High-pressure homogenization further modifies this interfacial organization, highlighting the central role of processing in defining the functional properties of rapeseed-based colloidal systems.

These findings provide new insight into the relationships between extraction conditions, interfacial composition and physicochemical stability. Rather than considering OBs and proteins as independent ingredients, this work supports the concept that their natural association constitutes a functional colloidal system whose behaviour can be tailored through the control of processing conditions and formulation parameters.

Overall, this study contributes to the development of green biorefinery strategies for rapeseed by combining sustainable extraction with the production of multifunctional food ingredients. It also establishes a scientific basis for engineering next-generation plant-based emulsions in which the architecture of the oil/water interface could be designed by exploiting the complementary functionalities of native OBs and storage proteins. Future work should investigate how processing variables, ionic environment and food matrix components modulate these interfacial interactions and evaluate their consequences on oxidative stability, digestion and sensory properties in complex food systems.

## CRediT authorship contribution statement

Hanitra Rabesona: Data curation, Formal analysis, Investigation, Methodology, Writing - review & editing. Christelle Lopez: Conceptualization, Supervision, Data curation, Formal analysis, Investigation, Methodology, Visualization, Validation, Project administration, Writing - review & editing.

## Funding

This research was funded by INRAE, project “plant oil bodies and proteins for innovative food emulsions” (Head of the research project: C. Lopez), and did not receive additional external funding.

## Declaration of competing interest

The authors declare that they have no known competing financial interests or personal relationships that could have appeared to influence the work reported in this paper.
